# Factors associated between behavior of administrating or recommending mumps vaccine and primary care physicians’ knowledge about vaccination: A nationwide cross‐sectional study in Japan

**DOI:** 10.1002/jgf2.471

**Published:** 2021-06-20

**Authors:** Jiro Takeuchi, Yuta Sakanishi, Tadao Okada, Kuniko Nakayama, Hiroshi Chiba, Rei Suganaga, Yosuke Nishioka, Tomomi Kishi, Tomio Suzuki, Preventive Medicine, Health Promotion Committee Vaccine Team, Japan Primary Care Association, Japan

**Affiliations:** ^1^ Clinical Epidemiology Hyogo College of Medicine Nishinomiya Japan; ^2^ Sakanishi Internal Medicine and Pediatrics Clinic Omuta Japan; ^3^ Tessyoukai Kameda Family Clinic Tateyama Tateyama Japan; ^4^ My family Clinic Gamagori Gamagori Japan; ^5^ Family Medical Practice Hanoi Hanoi Vietnam; ^6^ Nishioka Memorial Central Clinic Shima Japan; ^7^ Japan Baptist Hospital Kyoto Japan; ^8^ Department of General Medicine Osaka Medical and Pharmaceutical University Takatsuki Japan

**Keywords:** cross‐sectional study, mumps, nationwide study, physicians, primary care, vaccination

## Abstract

**Background:**

In Japan, the mumps‐containing vaccine was withdrawn from routine vaccination in 1993, and it became a voluntary vaccination. This study aimed to evaluate the association between the physicians’ knowledge about vaccinations and the administration or recommendation of the mumps vaccine.

**Methods:**

We conducted a nationwide cross‐sectional study targeting primary care physicians (PCPs) in Japan. We used a web‐based self‐administered questionnaire by Preventive Medicine and Health Promotion Committee Vaccine Team, the Japan Primary Care Association (JPCA), from March to June in 2019. The outcome of the study was the association between PCPs’ knowledge about vaccine and the administration or recommendation of mumps vaccine. We obtained the information on background, subsidies of mumps vaccination for children from the local government, and vaccination quiz scores. We performed logistic regression analysis to estimate the odds ratios (ORs) and 95% confidence intervals (CIs).

**Results:**

Among 10,470 PCPs in JPCA, 5075 were excluded. We received responses from 1084 PCPs (20.1%) and enrolled 981 participants in the analysis. PCPs with a higher score on the vaccination quizzes were significantly more likely to administrate the mumps vaccine for adults (adjusted odds ratio [AOR] 1.93, 95% CI 1.45–2.59, *p* < 0.001) and recommend mumps vaccine to adults than PCPs with a lower score (AOR 1.78, 95% CI 1.33–2.40, *p* < 0.001).

**Conclusions:**

We revealed an association between the administration or recommendation of mumps vaccine and PCPs’ better vaccination knowledge.

## 1 INTRODUCTION

The number of mumps infections in Japan in 2017 was 77,884; however, in the United States, where two mumps vaccinations are administered as regular immunization, it was only 6109.^1^ In Japan, at least 348 people were diagnosed with deafness caused by mumps infection during January 2015 to December 2016.^2^ It is important to immunize mumps vaccine not only for children but also for adults because there are reports of deafness by mumps infection in adulthood in Japan.^2^ Herd immunity with better mumps vaccination coverage or rate is required to reduce the frequency of deafness from mumps infection. We require 85–90% herd immunity against mumps infection.^3^

In Japan, the measles–mumps–rubella vaccine was regulated as a routine vaccine in 1989, just as in overseas countries. This was, however, discontinued in 1993 owing to an unexpectedly high prevalence of aseptic meningitis as an adverse effect of the vaccine; thus, the mumps vaccine was only provided as a single voluntary vaccination since then (measles–rubella combined vaccine remained as routine).^4^ In 2015, the vaccination rate remained only 30–40%.^5^ Therefore, the authors considered that the vaccination with the local government's subsidy could effectively improve the vaccination rate by reducing the economic burden. According to a national survey in 2020, 42% (full subsidy 115 and partial subsidy 361, out of 1125) of local governments subsidized the mumps vaccination.^6^

We searched mumps vaccination records of medical college students from 2008 to 2009 and found that only 58% of students (552 out of 949) received the mumps vaccine. In addition, perinatal history, past medical history, presence of siblings, and history of going abroad were not associated with mumps virus antibody positivity.^7^ It is necessary for physicians to change their behavior to vaccination. Six main factors specific to physician practice regarding vaccination are guideline implementation, characteristics of practice, laws and incentives, patient characteristics/problems, social norms, and knowledge and skills.^8^ After evaluating vaccine factors, we mainly focused on physician factors. Several researchers reported that recommendation by physicians affects recipients’ vaccine decisions.^10^, ^11^, ^12^, ^13^ Physicians’ appropriate knowledge about the vaccine is important for increasing vaccine administration.^14^, ^15^ Since there is no cost exemption or guidance from the government for voluntary vaccination, it is critical that it is recommended by a medical provider with appropriate knowledge. Nevertheless, the necessity of physicians’ knowledge for the administration or recommendation of mumps vaccine in Japan is unknown. Thus, the study aimed to clarify the association between physicians’ knowledge and the administration or recommendation of mumps vaccine.

## 2 METHODS

### 2.1 Study design, setting, and population

We conducted a cross‐sectional study among primary care physicians (PCPs) using a web‐based self‐administered questionnaire by Preventive Medicine and Health Promotion Committee Vaccine Team, the Japan Primary Care Association (JPCA), which is the largest academic association for PCPs in Japan. Most JPCA physicians were internists working as PCPs at a clinic or hospital. The survey was conducted from March to June 2019 and included only JPCA members.

### 2.2 Eligible Criteria

PCPs who were retired or were junior residents within 2 years after graduation from the medical school were excluded. This is because junior residents within 2 years cannot continuously work at outpatient vaccination. Further exclusion criteria included PCPs living outside Japan, those employed in a nonclinical setting, and those with missing data.

### 2.3 Questionnaire

Questionnaire items were revised from previous questionnaires used by the same team.^15^ We used an anonymous self‐administered questionnaire and collected data on the participating PCPs’ baseline characteristics such as gender, career after graduation, experience raising children, provision of daily pediatric medical service, provision of medical service at their main working facility (clinic, hospital, or other), the local government region of their main working facility, local government subsidies for mumps children vaccination, and vaccination quiz scores. We used web‐based self‐administered questionnaires by Preventive Medicine and Health Promotion Committee Vaccine Team, JPCA, distributed through the online mailing list of JPCA members.

### 2.4 Main outcome

This study's primary outcome was an association between primary care PCPs’ knowledge about vaccine and PCPs’ administration of mumps vaccine for adults. PCPs were asked the following yes–no question: “Do you administer mumps vaccination for adults?” The secondary outcome of this study was an association between PCPs’ knowledge about vaccine and PCPs’ recommendation of mumps vaccine for adults or children. PCPs were asked a multiple‐choice question with the following answers: “Actively recommend,” “Recommend occasionally,” “No opinion,” “Not actively recommend,” and “Not recommend.” Answers of “Actively recommend” were defined as “recommending behavior” based on the previous research, which is a more positive behavior.^10^ Furthermore, “Recommend occasionally,” “No opinion,” “Not actively recommend,” and “Not recommend” were defined as “no recommending behavior.” We obtained binary variables for the main outcome through these processes.

### 2.5 Main factor

The main factor was PCPs’ knowledge of vaccination, measured by a score on vaccination quizzes. The Preventive Medicine and Health Promotion Committee Vaccine Team from the JPCA prepared the quizzes by adopting the Delphi method.^16^ The quizzes comprised six general vaccine questions covering Japanese vaccination affairs, including a question about mumps vaccination. The score of the quizzes was the number of correct answers to each of the six questions. We set high score as near the top 50% for acquiring points or more. We set a low score as fewer points to obtain a binary variable. We set score on the vaccination quizzes (high/low) for independent variable.

### 2.6 Possible confounding factors

Possible confounding factors included experience raising children, career length after graduation (3–5 years, 6–10 years, 11–20 years, 21–30 years, 31–40 years, and ≥41 years), possession of any specialist qualifications, including primary care, information resources about vaccinations (government, academic, commercial,^17^ online professional community by the medical service provider, such as web site/Facebook group/Twitter/mailing list,^18^ and none), providing medical service at their main working facility, main working region (50,000 or more people), a high (≥10%) or low (<10%) proportion of pediatric patients in the total population, and local government subsidies for mumps vaccination of children (yes/no).^19^

### 2.7 Statistical analysis

We performed univariate and multivariate logistic regression analysis to estimate the odds ratios (ORs), the adjusted odds ratio(AORs), and 95% confidence intervals (CIs) using binary variables for the main outcome. We investigated the association between PCPs’ knowledge of vaccination and their administration or recommendation of mumps vaccine. We considered submitting one independent variable as possible confounding factors per 10 events in principle.^20^ In this case, we required the following variables: experience raising children, career length after graduation, possession of any specialist qualifications including primary care, information resources about vaccinations, providing medical service at their main working facility, main working region, a high or low proportion of pediatric patients in the total population, and local government subsidies for mumps vaccination of children. We then required at least 80 events for each primary or secondary outcome. We evaluated sensitivity analysis to inspect each variation for only mumps vaccination knowledge (correct or incorrect) instead of all the quizzes.

All statistical analyses were two‐tailed, with significance set at *p* < 0.05. The analysis was performed using Stata/SE 16.2 (StataCorp LLC, College Station, TX, USA).

### 2.8 Ethics

We obtained written informed consent from all participants before conducting the survey. The study protocol was approved by the Institutional Review Board at Osaka Medical College (Rin‐763).

## 3 RESULTS

### 3.1 Study flow and demographics

**FIGURE 1:**
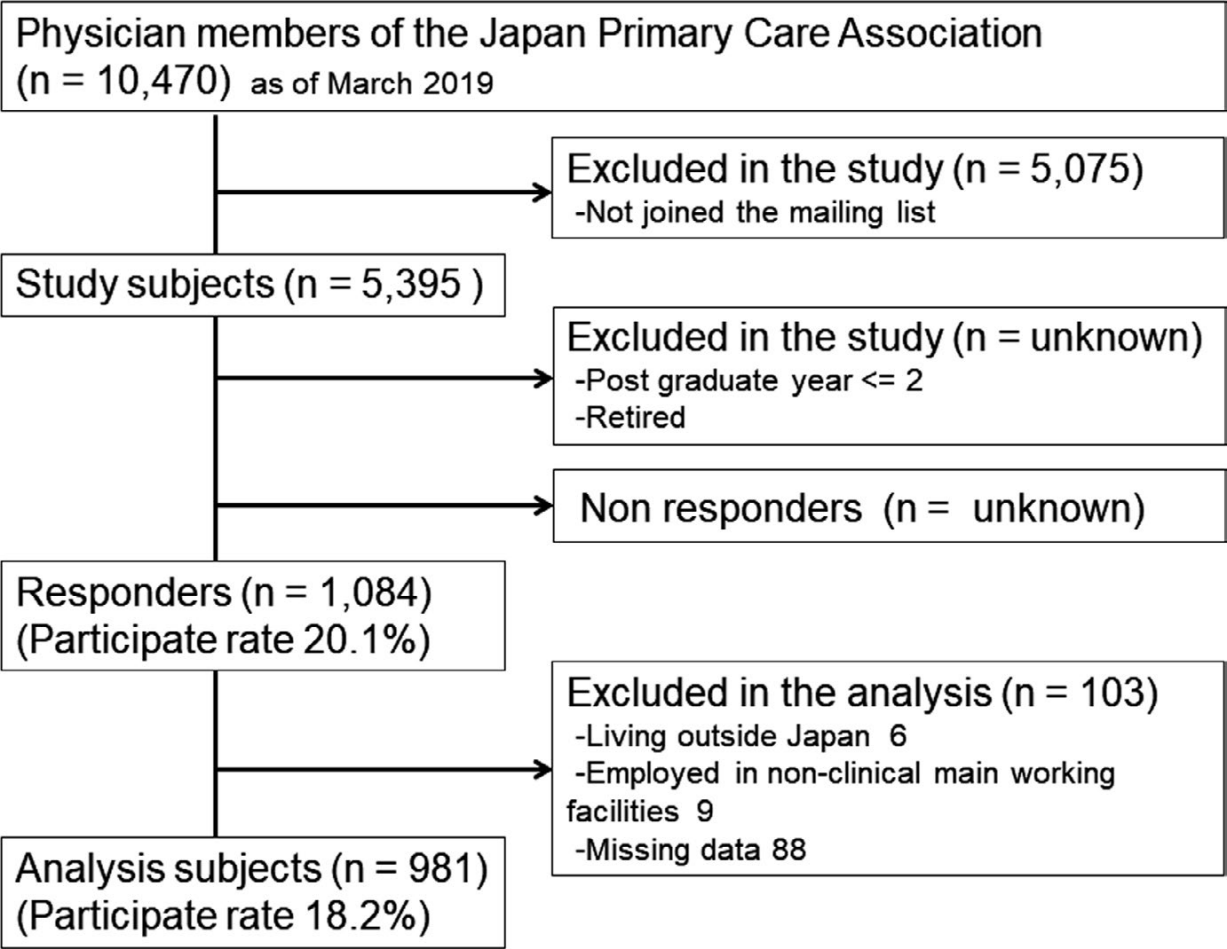
Study flow

Among 10,470 physicians in the JPCA, 5075 who did not join the mailing list were excluded. We received responses from 1084 of 5395 PCPs (response rate: 20.1%). The respondents were from all 47 prefectures of Japan. An additional 103 participants were excluded because they lived outside Japan, they performed nonclinical work, or they had missing data (Figure 1). The median (interquartile range) score on vaccination quizzes was 4 (2–5) points. The minimum and maximum scores were 0 and 6 points, respectively, and the mean score (standard deviation) was 3.47 (1.68) points. We set high score for 3 points or more. We set a low score as less than 3 points to obtain a binary variable. Then, participants’ baseline characteristics showed that 739 (75.3%) participants were males, 358 (36.5%) have been working for 11–20 years after graduation, 420 (42.8%) were mainly working in clinics, 378 (38.6%) were working in the suburbs, and 436 (44.4%) were working in a clinical setting where the proportion of pediatric patients on the patient panel was 0–10%. (Table 1).

**TABLE 1:** Characteristics of the participants

	Analysis subjects (n = 981)
Gender: male	739 (75.3)
Experience raising children	721 (73.5)
Postgraduate year	
3 y to 5 y	92 (9.4)
6 y to 10 y	178 (18.1)
11 y to 20 y	358 (36.5)
21 y to 30 y	193 (19.7)
31 y to 40 y	134 (13.7)
≥41 y	26 (2.6)
Main practice setting (n = 978)	
University hospital and general hospital	283 (28.5)
Other hospital	255 (25.7)
Clinic	420 (42.8)
University and laboratory center	16 (1.6)
Government and health service	4 (0.4)
Others	9 (0.9)
Main working region as administrative unit of local government	
Metropolitan area (≥300,000)	341 (34.8)
Suburb (<300,000 and ≥50,000)	378 (38.6)
Rural area (<50,000)	169 (17.3)
Remote area	91 (9.3)
A proportion of pediatric patients	
>30%	99 (10.1)
30% ≥ and >10%	184 (18.8)
10% ≥ and >0%	436 (44.4)
0%	262 (26.7)

Note

Values are expressed in numbers (percentage).

### 3.2 Factors associated with the administration of mumps vaccine for adults

PCPs with higher scores on the vaccination quizzes were significantly more likely to administer the mumps vaccine to adults than those with lower scores (adjusted odds ratio [AOR] 1.94, 95% CI 1.45–2.59, *p* < 0.001) (Table 2). There was also a positive association between voluntary mumps vaccination for adults and PCPs who were working 21–30 years after graduation (AOR 2.27, 95% CI 1.11–4.69, *p* = 0.03), those working 31–40 years after graduation (AOR 2.71, 95% CI 1.30–5.62, *p* = 0.008), those who worked in an urban area (AOR 1.37, 95% CI 1.0009–1.87, *p* = 0.049), those who had a higher proportion of pediatric patients (AOR 1.85, 95% CI 1.35–2.52, *p* < 0.001), those acquiring information from governments (AOR 1.98, 95% CI 1.40–2.81, *p* < 0.001), and those who had social network service or mailing list from an individual or group of medical service providers (AOR 1.55, 95% CI 1.14–2.11, *p* = 0.006).

**TABLE 2:** Factors associated with administrating behaviors of mumps voluntary vaccine for adults

	The group who vaccinates against mumps (n = 417) (42.5%)	The group who does not vaccinate against mumps (n = 564) (57.5%)	Crude odds ratio (95%CI) (n = 981)	*p*‐value	Adjusted odds ratio (95%CI) (n = 980)	*p*‐value
High scores in vaccination quiz (3 points and more over 6 points)	270 (64.8)	241 (42.7)	2.46 (1.90–3.20)	<0.001	1.94 (1.45–2.59)	<0.001
Gender (male)	324 (77.7)	415 (73.6)	1.25 (0.93–1.68)	0.14		
Having roaring experience	326 (78.2)	395 (70.0)	1.53 (1.14–2.06)	0.004	1.07 (0.76–1.50)	0.71
Career after graduation						
3 y to 5 y	20 (4.8)	72 (12.8)	Reference		Reference	
6 y to 10 y	62 (14.9)	119 (21.1)	1.92 (1.07–3.45)	0.03	1.33 (0.67–2.66)	0.42
11 y to 20 y	163 (39.1)	201 (35.6)	2.94 (1.72–5.04)	<0.001	1.89 (0.94–3.80)	0.07
21 y to 30 y	98 (23.5)	99 (17.6)	3.42 (1.93–6.05)	<0.001	2.27 (1.11–4.69)	0.03
31 y to 40 y	70 (16.8)	65 (11.5)	3.94 (2.16–7.18)	<0.001	2.71 (1.30– 5.62)	0.008
≥41 y	10 (2.3)	17 (3.0)	2.25 (0.89–5.72)	0.09	1.53 (0.54–4.34)	0.43
Possession of any specialist qualification (n = 980)	375 (89.9)	469 (83.3)	1.79 (1.21–2.64)	0.003	1.29 (0.78–2.15)	0.32
Providing medical service at main working facility	410 (98.3)	545 (96.6)	2.04 (0.85–4.90)	0.11	1.98 (0.77–5.10)	0.16
Mainly working in urban area (50,000 and more people as an administrative unit of local government)	320 (76.7)	399 (70.7)	1.36 (1.02–1.82)	0.04	1.37 (1.0009–1.87)	0.049
Providing daily pediatric medical service (≥10% of total patients)	158 (37.9)	125 (22.2)	2.14 (1.62–2.84)	<0.001	1.85 (1.35–2.52)	<0.001
Subsidy for mumps vaccination by local government for children	120 (28.8)	121 (21.5)	1.48 (1.10–1.98)	0.009	1.30 (0.94–1.78)	0.11
Information resource						
Government	349 (83.7)	398 (70.6)	2.14 (1.56–2.94)	<0.001	1.98 (1.40–2.81)	<0.001
Academia	399 (95.7)	514 (91.1)	2.16 (1.24–3.75)	0.007	1.17 (0.59–2.33)	0.66
Commerce	144 (34.5)	139 (24.7)	1.61 (1.22–2.13)	0.001	1.28 (0.93–1.75)	0.13
Social network service or mailing list by medical service providers of individuals or group	165 (39.6)	134 (23.8)	2.10 (1.59–2.77)	<0.001	1.55 (1.14–2.11)	0.006
None	6 (1.4)	14 (2.5)	0.57 (0.22 −1.51)	0.26	2.03 (0.59–6.92)	0.26

Note

As a result of adjusted all factors which we displayed by multivariate logistic regression analysis each other, each factor indicates an independent value for the adjustment odds ratio.

### 3.3 Factors associated with the recommendation of mumps vaccine for adults

The classification of PCP recommendation of mumps vaccine for adults showed that 327 PCPs “Actively recommend” (33.3%), 350 “Recommend occasionally” (35.7%), 223 “No opinion,” (22.7%), 54 “Not actively recommend” (5.5%), and 27 “Not recommend” (2.8%) (Table 3). PCPs with higher scores on the vaccination quizzes were significantly more likely to recommend mumps vaccination for adults than those with low scores (AOR 1.79, 95% CI 1.33–2.40, *p* < 0.001). No other factors were associated with the recommendation of mumps vaccination for adults.

**TABLE 3:** Factors associated with recommending behaviors to mumps vaccine for adults

	The group that recommends vaccine against mumps (n = 327) (33.3%)	The group that does not recommend vaccine against mumps (n = 654) (66.7%)	Crude odds ratio (95%CI) (n = 981)	*P*‐value	Adjusted odds ratio (95%CI) (n = 980)	*P*‐value
High scores in vaccination quiz (3 points and more over 6 points)	206 (63.0)	305 (46.6)	1.95 (1.48––2.56)	<0.001	1.79 (1.33–2.40)	<0.001
Gender (male)	254 (77.7)	485 (74.2)	1.21 (0.89–1.66)	0.23		
Having roaring experience	250 (76.5)	471 (72.0)	1.26 (0.93–1.72)	0.14	1.02 (0.72–1.44)	0.92
Career after graduation						
3 y to 5 y	23 (7.0)	69 (10.6)	Reference		Reference	
6 y to 10 y	50 (15.3)	128 (19.6)	1.17 (0.66–2.08)	0.59	1.10 (0.56–2.16)	0.79
11 y to 20 y	125 (38.2)	233 (35.6)	1.61 (0.96–2.71)	0.07	1.40 (0.71–2.76)	0.33
21 y to 30 y	71 (21.7)	122 (18.7)	1.75 (1.002–3.04)	0.049	1.55 (0.77–3.14)	0.22
31 y to 40 y	50 (15.3)	84 (12.8)	1.79 (0.99–3.21)	0.053	1.62 (0.79–3.30)	0.19
≥41 y	8 (2.5)	18 (2.8)	1.33 (0.51–3.47)	0.56	1.29 (0.45–3.66)	0.64
Possession of any specialist qualification (n = 980)	375 (89.9)	469 (83.3)	1.19 (0.80–1.76)	0.39	0.89 (0.54–1.47)	0.64
Providing medical service at main working facility	321 (98.2)	634 (96.9)	2.04 (0.85–4.90)	0.27	1.59 (0.61–4.14)	0.34
Mainly working in urban area (50,000 and more people as an administrative unit of local government)	252 (77.1)	467 (71.4)	0.85 (0.33–2.24)	0.06	1.35 (0.98–1.86)	0.06
Providing daily pediatric medical service (≥10% of total patients)	102 (31.2)	181 (27.7)	1.18 (0.89–1.58)	0.25	1.004 (0.74–1.37)	0.98
Subsidy for mumps vaccination by local government for children	84 (25.7)	157 (24.0)	1.09 (0.81–1.49)	0.56	1.01 (0.74–1.39)	0.94
Information resource						
Government	255 (78.0)	492 (75.2)	1.17 (0.85–1.60)	0.34	1.10 (0.78–1.53)	0.60
Academia	313 (95.7)	600 (91.7)	2.01 (1.10–3.68)	0.02	1.69 (0.79–3.64)	0.18
Commerce	104 (31.8)	179 (27.4)	1.24 (0.93–1.65)	0.15	1.11 (0.81–1.52)	0.51
Social network service or mailing list by medical service providers to individual or group	117 (35.8)	182 (27.8)	2.10 (1.59–2.77)	0.01	1.18 (0.87–1.61)	0.30
None	6 (1.8)	14 (2.1)	1.44 (1.09–1.92)	0.75	2.13 (0.62–7.32)	0.23

Note

As a result of adjusted all factors which we displayed by multivariate logistic regression analysis each other, each factor indicates an independent value for the adjustment odds ratio.

### 3.4 Factors associated with recommendation of voluntary mumps vaccine for children

The classification of PCP recommendation of mumps vaccination for children showed that 731 PCPs “Actively recommend” (74.5%), 168 “Recommend occasionally” (17.1%), 57 “No opinion,” (5.8%), 17 “Not actively recommend” (1.8%), and 8 “Not recommend” (0.8%) (Table 4).

**TABLE 4:** Factors associated with recommending behaviors of mumps voluntary vaccine for children

	The group that recommends vaccination against mumps (n = 731) (74.5%)	The group that does not recommend vaccination against mumps (n = 250) (25.5%)	Crude odds ratio (95%CI) (n = 981)	*p*‐value	Adjusted odds ratio (95%CI) (n = 980)	*p*‐value
High scores in vaccination quiz (3 points and more over 6 points)	429 (58.7)	82 (32.8)	2.91 (2.15–3.94)	<0.001	1.97 (1.41–2.75)	<0.001
Gender (male)	548 (75.0)	191 (76.4)	0.93 (0.66–1.30)	0.65		
Having roaring experience	543 (74.3)	178 (71.2)	1.17 (0.85–1.61)	0.34	1.12 (0.77–1.64)	0.55
Career after graduation						
3 y to 5 y	69 (9.4)	23 (9.2)	Reference		Reference	
6 y to 10 y	138 (18.9)	40 (16.0)	1.15 (0.64–2.07)	0.64	0.76 (0.37–1.55)	0.45
11 y to 20 y	289 (39.5)	69 (27.6)	1.40 (0.81–2.40)	0.23	0.84 (0.41–1.74)	0.64
21 y to 30 y	131 (17.9)	62 (24.8)	0.70 (0.40–1.23)	0.22	0.46 (0.22–0.97)	0.043
31 y to 40 y	85 (11.6)	49 (19.6)	0.58 (0.32–1.04)	0.07	0.40 (0.19–0.86)	0.02
≥41 y	19 (2.6)	7 (2.8)	0.90 (0.34–2.43)	0.84	0.83 (0.27–2.56)	0.74
Possession of any specialist qualification (n = 980)	632 (86.5)	212 (85.1)	1.11 (0.74–1.68)	0.60	1.33 (0.77–2.30)	0.31
Providing medical service at main working facility	717 (98.1)	238 (95.2)	2.58 (1.18–5.66)	0.02	1.82 (0.76–4.34)	0.18
Mainly working in urban area (50,000 and more people as an administrative unit of local government)	530 (72.5)	189 (75.6)	0.85 (0.61–1.19)	0.34	0.89 (0.62–1.27)	0.51
Providing daily pediatric medical service (≥10% of total patients)	248 (33.9)	35 (14.0)	1.17 (0.85–1.61)	<0.001	2.18 (1.44–3.29)	<0.001
Subsidy for mumps vaccination by local government for children	210 (28.7)	31 (12.4)	2.85 (1.89–4.29)	<0.001	2.42 (1.57–3.71)	<0.001
Information resource						
Government	561 (76.7)	186 (74.4)	1.14 (0.81–1.58)	0.45	1.03 (0.70–1.51)	0.87
Academia	698 (95.5)	215 (86.0)	3.44 (2.09–5.67)	<0.001	1.92 (1.01–3.63)	0.045
Commerce	209 (28.6)	74 (29.6)	0.95 (0.69–1.31)	0.76	0.86 (0.60–1.24)	0.42
Social network service or mailing list by medical service providers of individuals or group	251 (34.3)	48 (19.2)	2.20 (1.55–3.12)	<0.001	1.86 (1.26–2.75)	0.002
None	10 (1.4)	10 (4.0)	0.33 (0.14–0.81)	0.02	1.17 (0.37–3.74)	0.79

Note

As a result of adjusted all factors which we displayed by multivariate logistic regression analysis each other, each factor indicates an independent value for the adjustment odds ratio.

PCPs with higher scores on the vaccination quizzes were significantly more likely to recommend the mumps vaccine for children than those with low scores (AOR 1.97, 95% CI 1.41–2.75, *p* < 0.001). There was also a positive association between voluntary mumps vaccination for children and PCPs who had a higher proportion of pediatric patients (AOR 2.18, 95% CI 1.44–3.29, *p* < 0.001), those who works in the area where local subsidy is provided (AOR 2.42, 95% CI 1.57–3.71, *p* < 0.001), those who acquired information from academia (AOR 1.92, 95% CI 1.01–3.63, *p* = 0.045), and those who had social network service or mailing list for medical service from individual group providers (AOR 1.86, 95% CI 1.26–2.75, *p* = 0.002). However, there was a negative association between voluntary mumps vaccination for children and PCPs who were working for 21–30 years after graduation (AOR 0.46, 95% CI 0.22–0.97, *p* = 0.043) or 31–40 years after graduation (AOR 0.40, 95% CI 0.19–0.86, *p* = 0.02).

### 3.5 Sensitivity analysis

PCPs with higher scores on the vaccination quizzes were significantly more likely to administer mumps vaccination for adults than those with low scores (AOR 1.45, 95% CI 1.10–1.91, *p* = 0.008). There was a positive association between voluntary mumps vaccination for adults and PCPs who worked 11–20 years after graduation, contrary to a negative association in the conventional analysis (AOR 2.11, 95% CI 1.06–4.21, *p* = 0.04), those working 21–30 years after graduation (AOR 2.55, 95% CI 1.24–5.22, *p* = 0.01), those working 31–40 years after graduation (AOR 2.93, 95% CI 1.41–6.08, *p* = 0.004), those who had a higher proportion of pediatric patients (AOR 2.08, 95% CI 1.53–2.82, *p* < 0.001), those acquiring information from governments (AOR 2.03, 95% CI 1.44–2.88, *p* < 0.001), and those who had social network service or mailing list for medical service from individual group providers (AOR 1.68, 95% CI 1.24–2.28, *p* = 0.001). The result was no association, from a positive association in conventional analysis, between voluntary mumps recommendation for children and PCPs who were mainly working in urban area (AOR 1.36, 95% CI 0.9997–1.86, *p* = 0.050) from being positive.

PCPs with higher scores on the vaccination quizzes were significantly more likely to recommend mumps vaccination for adults than those with low scores (AOR 2.10, 95% CI 1.59–2.78, *p* < 0.001). None of factors were changed in significance with the recommendation of mumps vaccine for adults.

PCPs with higher scores on the vaccination quizzes were significantly more likely to recommend mumps vaccine for children than those with low scores (AOR 2.25, 95% CI 1.63–3.11, *p* < 0.001). There was also a positive association between voluntary mumps vaccination for children and PCPs who had a higher proportion of pediatric patients (AOR 2.57, 95% CI 1.71–3.87, *p* < 0.001), those who works in the area where local subsidy is provided (AOR 2.44, 95% CI 1.59–3.75, *p* < 0.001), those who acquired information from academia (AOR 2.18, 95% CI 1.15–4.12, *p* = 0.02), and those who had social network service or mailing list for medical service from individual group providers (AOR 1.99, 95% CI 1.35–2.93, *p* = 0.001). None of factors were changed in significance with the recommendation of mumps vaccine for children.

## 4 DISCUSSION

We conducted this survey among physician members of the JPCA to assess Japanese PCPs’ characteristics. We found positive associations between PCPs’ knowledge of vaccination and the administration or recommendation of mumps vaccine. We also found positive associations between certain information resources and the administration or recommendation of mumps vaccine.

Based on our results, PCPs should acquire better knowledge about vaccination to improve vaccination rates in both adults and children. Members of the JPCA can attend The Vaccine Seminar as an independent seminar, the Lifelong Education Seminar of the JPCA, and other events to improve their vaccine knowledge.^21^ Information resources from the online professional community by medical service providers seem to be associated with administration of voluntary mumps vaccine for adults and recommendation of voluntary mumps vaccine for adults and recommendation of vaccine for children. This could be based on PCPs’ interest in how other doctors recommend and administer the vaccination.^22^ Acquiring information of other appropriately practicing PCPs may increase mumps vaccine administration. PCPs may also want to gather scientific evidence about mumps vaccination, which could be why academic information is associated with the recommendation to voluntary mumps vaccine for children. Academic information provides both PCPs and vaccination candidates with knowledge based on scientific evidence, increasing providers’ tendency to recommend and patient willingness to receive vaccines.

The number of years working after graduation was also associated with the administration or recommendation of voluntary mumps vaccine for adults and children, respectively. It is speculated that PCPs working 21–40 years after graduation are important in increasing the vaccination rate. However, a paradox existed in our results, that is, the range of the working years after graduation was positively associated with the administration of voluntary mumps vaccine for adults but negatively associated with the recommendation of voluntary mumps vaccine for children. Sakanishi et al. reported generation‐specific gaps regarding vaccination recommendation in 2012, which described PCPs working 3–10 years after graduation as more disposed to recommended Haemophilus influenza type b vaccine, seven‐valent pneumococcal conjugate vaccine, and human papillomavirus vaccine than PCPs working 11–40 years after graduation.^14^ On the other hand, PCPs with a higher postgraduate year may generally have a greater interest in mumps vaccines at the present study. PCPs working 21–40 years after graduation might tend to administer voluntary mumps vaccine for adults and recommend voluntary mumps vaccine for children because they might be impressed with the initiation of routine mumps vaccination in 1989 as their sensitive periods after graduation in 1989 to 1998.^4^ These results need to reiterate the importance of credible information about vaccination to maintain and update PCP’s knowledge for all generations.

The tendency to administer voluntary mumps vaccination for adults requires not only knowledge about vaccination but also knowledge about daily pediatric medical care. The main target for voluntary mumps vaccination is children, and PCPs acquire more experience in administering mumps vaccine to children. As a result, PCPs tend to administer mumps vaccinations for adults. This indicates that both knowledge and experience are necessary to increase vaccination rates.

Local societal factors are also associated with voluntary mumps vaccination. We observed that a higher proportion of pediatric patients was not associated with the tendency to recommend voluntary mumps vaccination for adults. However, a higher proportion of pediatric patients was associated with an increased tendency to recommend voluntary mumps vaccines for children. Adult vaccine candidates in urban areas are potentially more likely to request mumps vaccination because they can access more information. If local governments subsidize voluntary mumps vaccination for children, the economic burden will be lower for vaccine recipients, making it easier for PCPs to recommend the vaccine. Subsidies also have the potential to improve vaccination rates.^15^, ^23^ The Japanese government subsidized the measles–rubella vaccine for a catch‐up campaign during a measles outbreak from 2008 to 2013.^24^ Furthermore, the local government's role would be greater because PCPs rely on voluntary mumps vaccine information from the local government.

We performed a sensitivity analysis to inspect each variation for the only mumps vaccination knowledge. We found almost no changes in results, especially the association between administration or recommendation of mumps vaccine and PCPs’ vaccination knowledge. It suggests that full vaccination knowledge could improve PCPs’ tendency to administer or recommend mumps vaccine as much as the only mumps vaccination knowledge. The results remain robust, and then, a brief statement to this effect must suffice.

This study had some limitations. First, there was selection bias owing to the low response rate. A higher proportion of PCPs in this study were younger than those in the total PCP population. Therefore, PCPs who actively promoted vaccination may have responded. This made it difficult to conclude about the overall option of the collective PCP group regarding vaccine administration or recommendation. Second, we did not investigate the administration of voluntary mumps vaccine in children. Therefore, we could not symmetrically discuss the relationships between adults and children, as well as the administration or recommendation of vaccination using a two‐by‐two matrix. We aimed to address this logically; however, further study is required to make accurate comparisons. Third, we could not completely investigate the factors contributing to administration or recommendation of mumps vaccine owing to the cross‐sectional nature of the study. Further studies are needed to confirm the proposed causality of each factor discussed in this study. Fourth, we did not adjust the effects of unknown confounding factors, which is a general limitation of observational studies. Finally, we could not obtain information from 5,075 PCPs because they did not join the mailing list. The loss of participants led to decreased sample size. Furthermore, there might be a higher selection bias in that those JPCA members who joined the mailing list might be interested in vaccination.

## 5 CONCLUSIONS

We revealed a significant association between PCPs’ better knowledge about vaccination and the administration or recommendation mumps vaccine. Our results suggest that providing more knowledge of vaccination to PCPs may increase their likelihood to administration or recommendation of mumps vaccine and improve vaccination rate.

## CONFLICT OF INTEREST

None declared.
